# Optimized Ultrasound-Assisted Extraction Enhances the Recovery of Anti-Collagenase Phytochemicals from *Mitragyna speciosa* Korth. Leaves

**DOI:** 10.3390/plants15162416

**Published:** 2026-08-07

**Authors:** Thasang Thavanapong, Nara Yaowiwat, Siripat Chaichit, Mathukorn Sainakham, Pimporn Leelapornpisid, Worrapan Poomanee

**Affiliations:** 1 Department of Pharmaceutical Sciences, Faculty of Pharmacy, Chiang Mai University, Chiang Mai 50200, Thailand; thasang_tha@cmu.ac.th (T.T.); siripat.chaichit@cmu.ac.th (S.C.); mathukorn.s@cmu.ac.th (M.S.); pim_leela@hotmail.com (P.L.); 2 Doctoral Degree Program in Toxicology, College of Pharmacy, Kaohsiung Medical University, Kaohsiung 80708, Taiwan; 3 School of Cosmetic Science, Mae Fah Luang University, Chiang Rai 57100, Thailand; nara.nan@mfu.ac.th; 4 Green Cosmetic Technology Research Group, School of Cosmetic Science, Mae Fah Luang University, Chiang Rai 57100, Thailand; 5 Laboratory for Molecular Design and Simulation (LMDS), Faculty of Pharmacy, Chiang Mai University, Chiang Mai 50200, Thailand; 6 Innovation Center for Holistic Health, Nutraceuticals, and Cosmeceuticals, Faculty of Pharmacy, Chiang Mai University, Chiang Mai 50200, Thailand

**Keywords:** *Mitragyna speciosa*, ultrasound-assisted extraction, response surface methodology, rutin, chlorogenic acid, antioxidant activity, collagenase inhibition, cosmeceutical, nutraceutical

## Abstract

*Mitragyna speciosa* Korth. (Kratom) is a plant native to Southeast Asia, traditionally used for its stimulant and analgesic properties. Beyond mitragynine, the principal alkaloid dominating *M. speciosa* research, this study explored the cosmeceutical and nutraceutical potential of its phenolic and flavonoid constituents. Kratom leaf extract (MLE) was obtained via ultrasound-assisted extraction optimized by Central Composite Design, yielding high polyphenol content and antioxidant capacity. The optimized MLE showed measurable collagenase inhibition (IC_50_ = 92.63 ± 7.81 µg/mL) while maintaining fibroblast viability (>80%). UHPLC-ESI-QTOF-MS/MS tentatively identified chlorogenic acid, rutin, and kaempferol 3-glucosyl-(1→6)-galactoside (K3G). Chlorogenic acid and rutin were quantified by HPLC at 31.37 ± 2.41 and 18.76 ± 1.66 µg/mg extract, respectively, while K3G remained linked to an unidentified peak. Mitragynine was also quantified at an appreciable level (59.38 ± 0.21 µg/mg extract), indicating that removal of the alkaloid fraction is required before application. Molecular docking identified chlorogenic acid as the strongest predicted collagenase binder (−12.82 kcal/mol), though chlorogenic acid and rutin together could not fully account for the extract’s activity, suggesting possible contributions from K3G and other unidentified compounds. These findings provide preliminary evidence for the cosmeceutical potential of kratom leaf extract, pending control of its alkaloid content.

## 1. Introduction

*Mitragyna speciosa* Korth. (Kratom) is a medicinal plant native to Southeast Asia with a long history of traditional use in folk medicine across Thailand, Malaysia, and neighboring countries, where its leaves have been traditionally consumed for their stimulant and analgesic properties, as well as in the management of fever, pain, and gastrointestinal conditions [1]. The economic significance of this plant was further elevated following the regulatory revision in Thailand in 2021, which reclassified it as an economic plant, creating new opportunities for its systematic scientific investigation and value-added utilization [2]. Among the most promising avenues is the exploitation of its leaves as a source of cosmeceutical ingredients, given that plant-derived resources have gained increasing attention for skin-related applications owing to their diverse phytochemical compositions and broad spectrum of biological activities [3]. Although indole alkaloids, particularly mitragynine, represent the most abundant and extensively studied constituents of *M. speciosa*, their well-documented dose-dependent toxicity and opioid receptor-mediated pharmacological activity make them less desirable for cosmeceutical development. In contrast, the phenolic and flavonoid constituents of the plant offer greater potential for such applications, as they are widely recognized as major antioxidant compounds and have been extensively studied for their roles in skin protection and anti-aging potential [4,5]. Previous studies have reported that *M. speciosa* leaves contain various phenolic and flavonoid compounds associated with notable antioxidant activity, suggesting their potential as a natural source of cosmeceutical ingredients [6]. However, despite increasing reports on its phytochemical composition, the skin anti-aging properties of *M. speciosa* leaf extracts, particularly those relevant to collagenase inhibition, have never been investigated, and the phytochemical class responsible for driving its anti-aging potential remains unidentified.

Skin aging is a complex biological process characterized by progressive structural and functional deterioration resulting from both intrinsic factors and extrinsic stressors such as chronic UV exposure, manifested clinically as wrinkles and reduced mechanical properties of the skin [7,8]. At the molecular level, oxidative stress is a crucial contributor to skin aging, primarily through the disruption of the extracellular matrix. Excessive levels of reactive oxygen species (ROS) induce the expression of matrix metalloproteinases (MMPs), including collagenase, thereby promoting collagen breakdown and compromising dermal structural integrity [9]. Given the growing demand for plant-derived cosmeceutical ingredients with multi-targeted anti-aging properties, *M. speciosa*, with its rich flavonoid and phenolic profile, represents a promising but underexplored candidate.

The functional quality of plant extracts is strongly influenced by the extraction process, as extraction conditions directly determine both the yield and the phytochemical composition of bioactive compounds [10]. Conventional extraction methods often require prolonged processing times, which may compromise the stability of thermolabile phytochemicals. Ultrasound-assisted extraction (UAE) has therefore been widely adopted as an efficient alternative, as acoustic cavitation enhances solvent penetration and disrupts plant cell walls, thereby improving extraction efficiency while reducing processing time [11,12]. Nevertheless, UAE performance is highly dependent on operating parameters, and systematic optimization is required to obtain extracts with desirable functional characteristics [13]. Central composite design (CCD)-based response surface methodology provides a robust statistical framework for evaluating extraction variables and identifying optimal conditions and has been widely applied in both extraction optimization and pharmaceutical development [14,15].

In this study, a systematic investigation integrating extraction optimization, phytochemical characterization, biological activity evaluation, and molecular docking analysis was conducted to provide scientific evidence for the cosmeceutical applicability of *M. speciosa* leaves. UAE conditions were optimized using a CCD, and the optimized extract was evaluated for antioxidant and anti-aging properties, including collagenase inhibitory activity supported by molecular docking, and fibroblast safety assessment. These findings contribute to the scientific foundation for the cosmeceutical development of this traditionally used medicinal plant as a value-added natural ingredient.

## 2. Results

### 2.1. Response Surface Methodology of UAE of M. speciosa Leaves

In this study, UAE of *M. speciosa* leaves was optimized using response surface methodology (RSM) with a CCD, employing three independent variables: liquid-to-solid ratio (A, mL/g), extraction time (B, min), and amplitude (C, %). Five responses were evaluated for each extract: extraction yield (Y_1_), total phenolic content (TPC, Y_2_), total flavonoid content (TFC, Y_3_), IC_50_ of DPPH (Y_4_), and EC_1_ of FRAP (Y_5_), as summarized in Table 1. Potential outliers were identified using normal Studentized residual plots. Runs that exhibited substantial deviations from linearity were excluded prior to model construction to improve residual normality and overall robustness [16].

As presented in Table 1, the extraction yield of *M. speciosa* leaves ranged from 4.29–10.63%. TPC and TFC varied between 112.65–238.53 mg GAE/g and 205.67–432.99 mg QE/g, respectively. Antioxidant capacity, expressed as IC_50_ (DPPH) and EC_1_ (FRAP), ranged from 23.92–77.41 µg/mL and 495.47–1588.17 µg/mL, respectively.

**Figure 1:**
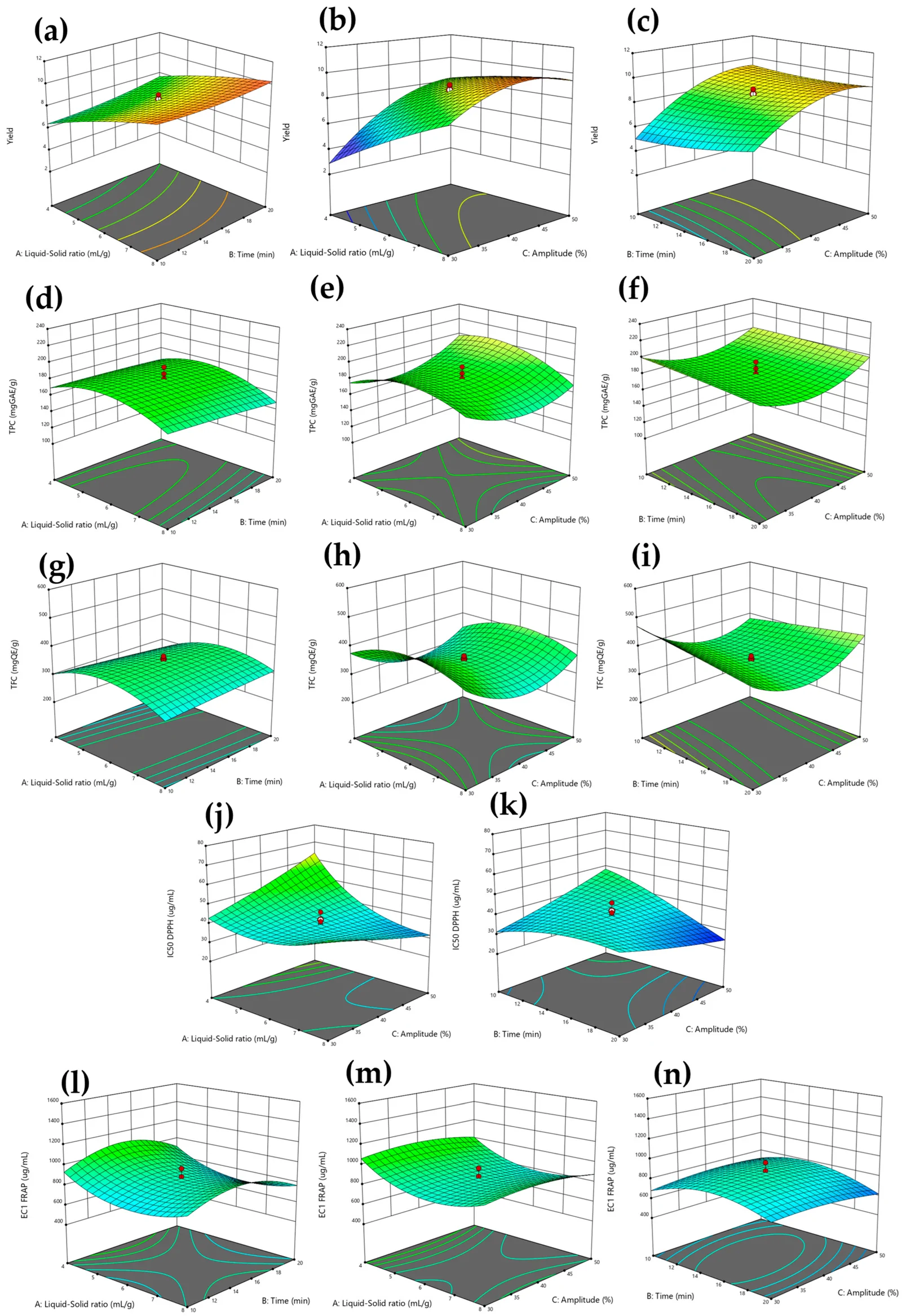
Response surface diagram showing (**a**–**c**) the interaction effects on extraction yield, (**d**–**f**) total phenolic content (TPC), (**g**–**i**) total flavonoid content (TFC), (**j**,**k**) IC_50_ of DPPH radical scavenging activity, and (**l**–**n**) EC_1_ of ferric reducing antioxidant power (FRAP) as a function of liquid-solid ratio, extraction time, and amplitude.

For each response, a few runs were excluded as outliers based on Studentized residual analysis: two runs for extraction yield (Runs 12, 13), one run for TPC (Run 12), two runs for TFC (Runs 12, 14), three runs for IC_50_ (DPPH) (Runs 3, 6, 7), and two runs for EC_1_ (FRAP) (Runs 10, 11). Following the exclusion of outliers, ANOVA confirmed that all models achieved the adequacy criteria (Table 2). This exclusion was justified by comparing model diagnostics before and after adjustment (Supplementary Table S1): The original models showed negative predicted R^2^ values (except for EC_1_) and adjusted–predicted R^2^ differences exceeding 0.2 across all five responses, with the yield model additionally showing significant lack-of-fit (*p* = 0.0185), whereas predicted-versus-actual plots (Supplementary Figure S1) showed a markedly improved linear fit after adjustment. The final models demonstrated high goodness-of-fit (R^2^ > 0.90), significant model *p*-values (<0.001), and non-significant lack-of-fit (*p* > 0.05). Moreover, the differences between adjusted R^2^ and predicted R^2^ were less than 0.2, and adequate precision values exceeded 4, satisfying the reliability criteria recommended by Design-Expert^®^ Version 10.0. (Stat-Ease, Minneapolis, MN, USA). The response surface diagrams (RSDs) for each response are presented in Figure 1.

### 2.2. Effects of Extraction Variables on Extraction Responses

#### 2.2.1. Extraction Yield

Based on the ANOVA results (Table 2), the significant terms included the linear effects (A, B, C), the interaction terms (AC, BC), and the quadratic term of amplitude (C^2^), yielding the following regression model:

%yield = 8.61 + 1.55A + 0.38B + 1.75C − 0.25AB − 0.95AC − 0.42BC − 0.24A^2^ + 0.25B^2^ − 1.28C^2^ (1)

Among the single factors, the liquid-to-solid ratio (A) and the ultrasound amplitude (C) both exhibited significant positive linear effects on extraction yield. The amplitude additionally showed a significant negative quadratic effect (C^2^), indicating a curvature response in which yield reached a plateau at higher amplitude levels (around 40%). For the interaction effects, AC and BC were both significantly negative.

#### 2.2.2. Phytochemical Recovery

The regression models for TPC and TFC are presented below:

TPC = 179.53 − 6.04A − 4.15B + 3.34C − 2.04AB − 7.94AC + 2.5BC − 16.69A^2^ − 0.26B^2^ + 18.64C^2^ (2)

TFC = 349 + 8.35A − 4.12B − 13.09C + 13.67AB + 1.34AC + 30.83BC − 54.58A^2^ − 1.43B^2^ + 76.02C^2^ (3)

For TPC, significant effects were observed for the liquid-to-solid ratio (A, A^2^), the quadratic term of amplitude (C^2^), and the AC interaction. The liquid-to-solid ratio showed significant negative linear and quadratic effects. The amplitude (C^2^) exhibited a significant positive quadratic effect, while the AC interaction term was significantly negative.

For TFC, significant terms included A^2^, C^2^, and the BC interaction. The quadratic term of A was significantly negative, whereas C^2^ was significantly positive. The BC interaction showed a significant positive effect.

#### 2.2.3. Antioxidant Activity

The regression models for IC_50_ and EC_1_ are as follows:

log_10_ [IC_50_] = 1.59 − 0.063A − 0.048B − 0.001C − 0.088AC − 0.092BC + 0.068A^2^ − 0.045B^2^ (4)

log_10_ [EC_1_] = 2.93 − 0.031A − 0.011B − 0.011C − 0.006AB − 0.004AC − 0.021BC + 0.078A^2^ − 0.075B^2^ − 0.023C^2^ (5)

For IC_50_ (DPPH), the linear terms of the liquid-to-solid ratio (A) and the extraction time (B) were significantly negative. The quadratic term A^2^ was significantly positive, while B^2^ was significantly negative. The interaction terms AC and BC were both significantly negative. The linear term of the amplitude (C) was not significant.

For EC_1_ (FRAP), the linear term of liquid-to-solid ratio (A) was significantly negative, following the same directional pattern as observed for IC_50_. Similarly, the quadratic term A^2^ was significantly positive and B^2^ was significantly negative, consistent with the IC_50_ model. In contrast to IC_50_, no significant terms involving the amplitude (C), whether linear, quadratic, or interaction, were identified in the EC_1_ model.

### 2.3. Optimized Condition of M. speciosa Leaves

The optimal conditions for UAE of *M. speciosa* leaves were determined by numerical optimization using Design-Expert software to maximize extraction yield (Y_1_), and phytochemical recovery (TPC, Y_2_), and total flavonoid content (TFC, Y_3_), while minimizing IC_50_ (Y_4_) and EC_1_ (Y_5_). The optimized parameters were a liquid-solid ratio of 6.355 mL/g, an extraction time of 20 min, and an amplitude of 50%. Model validation was performed in triplicate under these conditions, and the predicted and experimental values (Table 3) showed close agreement, with %RSE values below 5% for all responses, indicating good predictive accuracy and model reliability within the studied range. The extract obtained under these optimized conditions was designated as MLE for subsequent analyses.

### 2.4. Phytochemical and Antioxidant Correlation

All experimental responses were statistically analyzed to examine the correlation between phytochemical contents (TPC and TFC) and biological activities (DPPH IC_50_ and EC_1_) of the *M. speciosa* leaf extract (Table 4). TFC showed a significant moderate negative correlation with both IC_50_ and EC_1_, indicating enhanced antioxidant potential with increasing flavonoid content. In contrast, TPC was not significantly correlated with antioxidant activity (*p* > 0.05). It should be noted that the TFC assay is not entirely specific to flavonoid. TFC-associated compounds encompassing both flavonoids and catechol-bearing phenolics were therefore prioritized for further investigation.

### 2.5. Phytochemical Profile

Table 5 presents the phytochemical constituents identified in the optimized *M. speciosa* leaf extract (MLE) by UHPLC-ESI-QTOF-MS/MS. A total of 13 compounds were tentatively characterized and classified into seven chemical groups, including alkaloids (5 compounds), flavonoids (2 compounds), phenolic acids (1 compound), triterpenoids (1 compound), glycosides (1 compound), sphingosides (1 compound), and organic acids (2 compounds). Alkaloids represented the numerically predominant class. All identified compounds satisfied the acceptance criteria of match score > 80% and low mass error values (within ±5 ppm), confirming high analytical reliability. However, the majority of these match scores were obtained from the accurate-mass PCD (Personal Compound Database), which reflects agreement in molecular formula and isotopic pattern only, without confirmation by reference MS/MS spectra; these nine compounds were therefore assigned an identification confidence of Level 3 (tentative candidate) according to Schymanski et al. [17]. In contrast, mitragynine, chlorogenic acid, rutin, and quinic acid were matched against the PCDL (Personal Compound Database and Library), which additionally incorporates reference MS/MS spectral data, and were accordingly assigned a higher confidence of Level 2a (probable structure). The characteristic MS/MS fragment ions supporting each compound annotation are provided in Supplementary Table S2. Notably, Pearson correlation analysis demonstrated a significant association between TFC and antioxidant activity. Among the four compounds identified with Level 2a confidence, only chlorogenic acid and rutin possess catechol/flavonoid moieties relevant to the TFC assay response, whereas mitragynine and quinic acid do not contribute to this signal. Chlorogenic acid and rutin were therefore selected for quantitative determination by HPLC to establish a direct, marker-based link between phytochemical content and the antioxidant activity of MLE.

### 2.6. Quantitative Determination of Chlorogenic Acid and Rutin in the MLE

**Figure 2:**
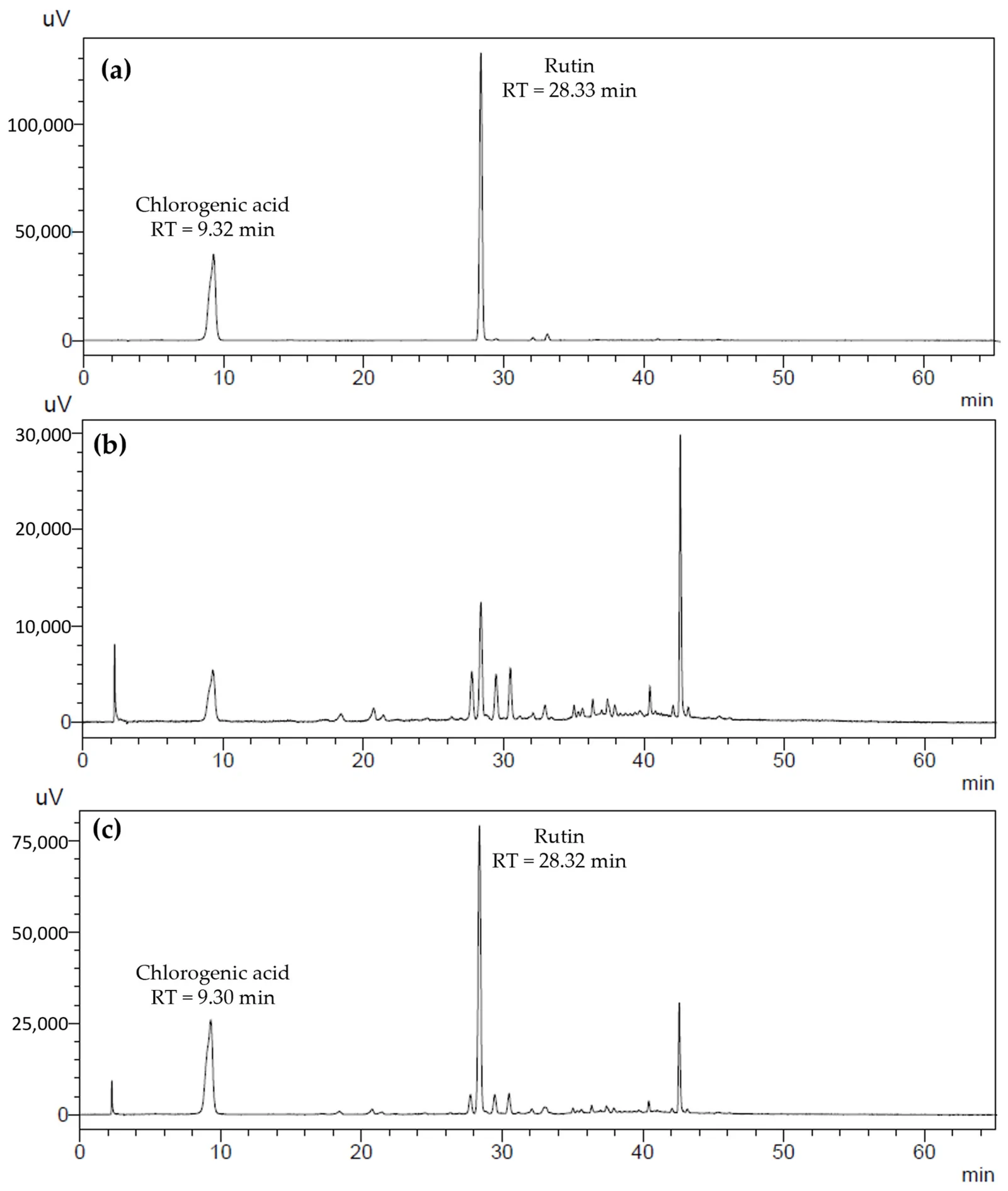
HPLC chromatogram of (**a**) standard mixture at 50 µg/mL, (**b**) MLE at 250 µg/mL, (**c**) MLE at 250 µg/mL spiked with standards at 25 µg/mL.

Chlorogenic acid and rutin, identified by UHPLC-QTOF-MS/MS, were quantified in the MLE by HPLC-UV. Calibration curves (1–100 µg/mL) showed excellent linearity (R^2^ > 0.999; Table 6). Both compounds were detected at retention times consistent with their authentic standards and were further confirmed by the standard addition method (Figure 2), elevating their identification confidence to Level 1 [17]. Quantification revealed 31.37 ± 2.41 and 18.76 ± 1.66 µg/mg extract of chlorogenic acid and rutin, respectively (Table 7), corresponding to approximately 3.1% and 1.8% (*w*/*w*) of MLE. In addition, mitragynine, the principal alkaloid of *M. speciosa*, was quantified to provide supporting safety-related information on the extract, using an authentic standard and a separate validated HPLC method (Table 6 and Table 7). It was quantified at 59.38 ± 0.21 µg/mg extract, corresponding to approximately 5.9% (*w*/*w*) of the MLE. Because the chromatographic conditions used for mitragynine differed from those applied to the phenolic and flavonoid constituents, its chromatogram is provided separately in Supplementary Figure S2.

The MLE chromatogram also exhibited several unidentified peaks, including a prominent peak at 41.32 min that could not be assigned to any of the compounds tentatively identified by UHPLC-QTOF-MS/MS (Table 5) and therefore remains uncharacterized. Among the tentatively identified metabolites, kaempferol 3-glucosyl-(1→6)-galactoside (K3G) possesses a flavonoid chromophore compatible with UV detection under the analytical conditions employed in this study. Based on its expected polarity, K3G would likely elute in close proximity to rutin and may correspond to one of the minor peaks observed in this region, suggesting its possible presence in the extract. This tentative annotation (Level 3 confidence) served solely as a scientific rationale for including K3G in the subsequent molecular docking analysis. However, as K3G was neither isolated, quantified, nor experimentally confirmed, its occurrence in the extract and its potential contribution to the observed biological activities remain speculative and warrant further experimental verification.

### 2.7. Cytotoxicity on Human Dermal Fibroblasts

**Figure 3:**
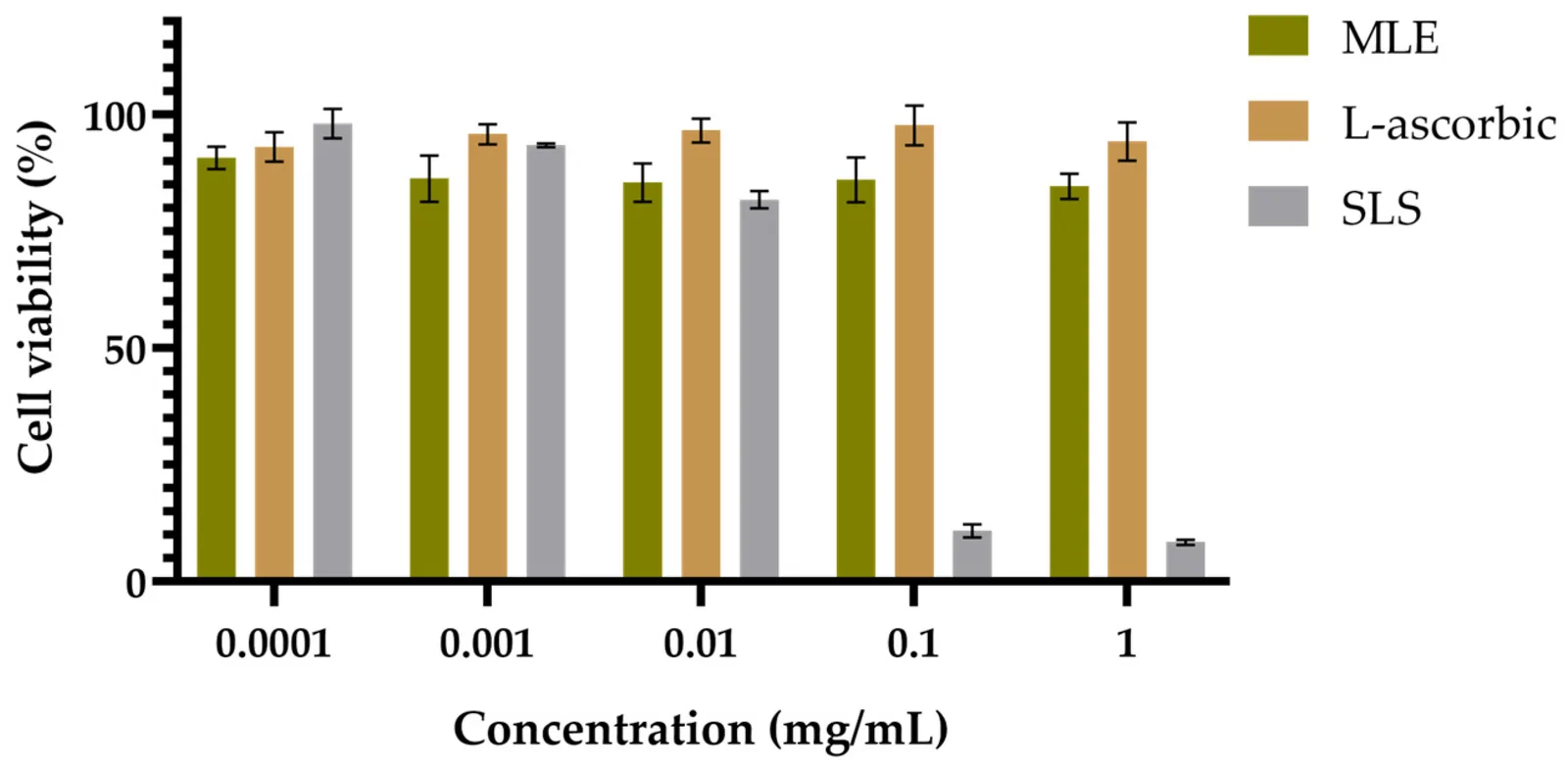
The percentage of cell viability in SRB cytotoxicity assay.

The cytotoxicity of the MLE on normal human dermal fibroblasts (NHDF) was evaluated and compared with L-ascorbic acid and sodium lauryl sulfate (SLS) (Figure 3). Both MLE and L-ascorbic acid showed no cytotoxicity across 0.0001–1 mg/mL, maintaining cell viability above 80%. At 1 mg/mL, viability was 84.66 ± 2.74% and 94.26 ± 4.10%, respectively. In contrast, SLS, which is a positive control, markedly reduced viability to 8.41 ± 0.53% at the same concentration.

### 2.8. Antioxidant and Collagenase Enzyme Inhibition

**Figure 4:**
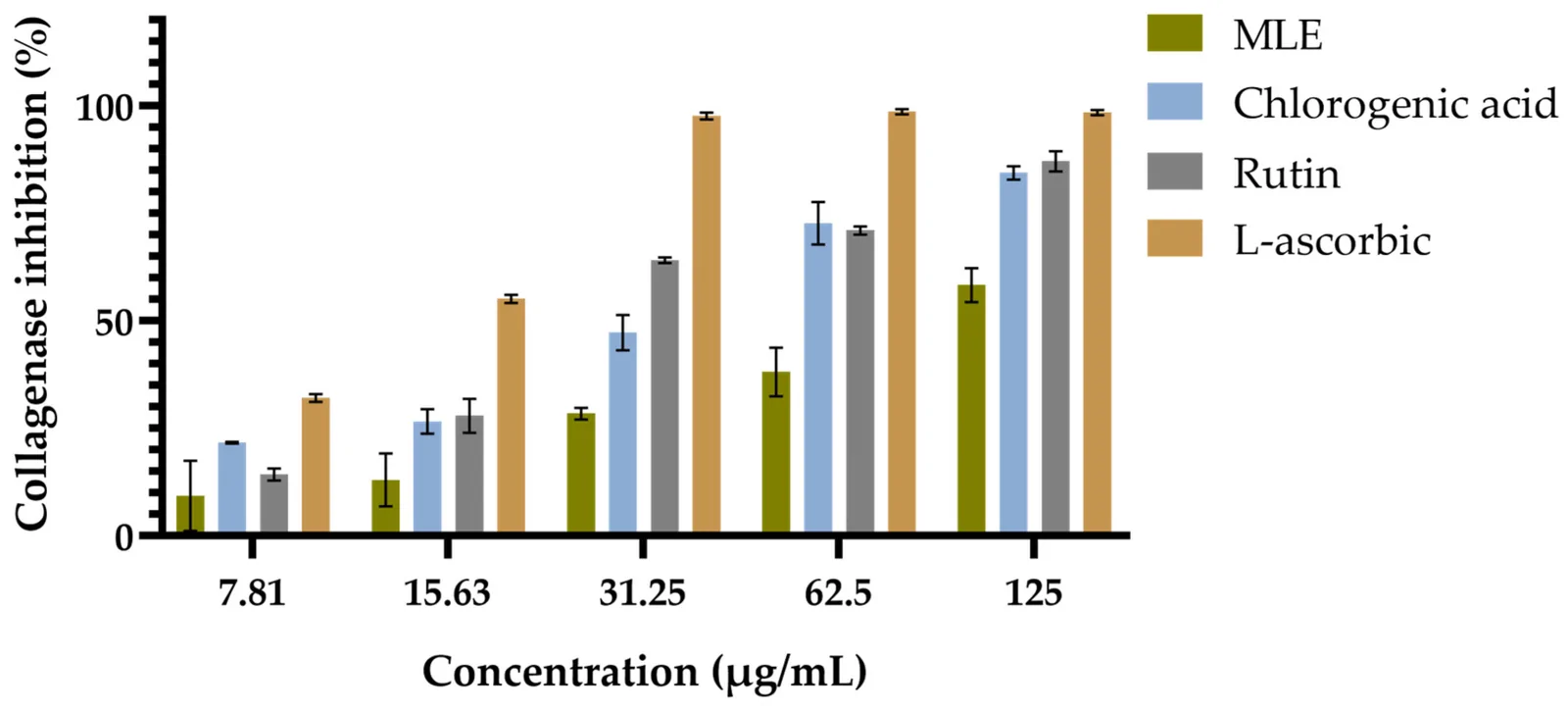
Inhibitory effects of the MLE, Chlorogenic acid, Rutin, and L-ascorbic acid in various concentrations (7.81–125 µg/mL) on collagenase enzyme (*n* = 3).

The antioxidant and anti-collagenase activities of MLE, chlorogenic acid, rutin, and L-ascorbic acid (positive control) were evaluated by DPPH, FRAP, and collagenase inhibition assays (Table 8; Figure 4). In both the DPPH and FRAP assays, MLE showed significantly weaker activity than the three individual compounds. Chlorogenic acid, rutin, and L-ascorbic acid did not differ significantly in DPPH scavenging activity; however, in the FRAP assay, chlorogenic acid was comparable to L-ascorbic acid, while rutin showed significantly lower reducing power than both. A similar pattern was observed for collagenase inhibition, where MLE was significantly less active than the individual compounds. Chlorogenic acid was significantly less potent than L-ascorbic acid, whereas rutin did not differ significantly from either chlorogenic acid or L-ascorbic acid.

### 2.9. Binding Affinity of the Bioactive Constituents on Collagenase Enzymes

Kaempferol 3-glucosyl-(1→6)-galactoside (K3G), a flavonoid glycoside identified in the phytochemical profile, was retained as an additional comparator in the docking analysis, as it remained the most plausible candidate among the profiled constituents. Since chlorogenic acid and rutin alone could not fully account for the anti-collagenase activity of MLE, K3G was included to explore its possible contribution to this unexplained activity.

**Figure 5:**
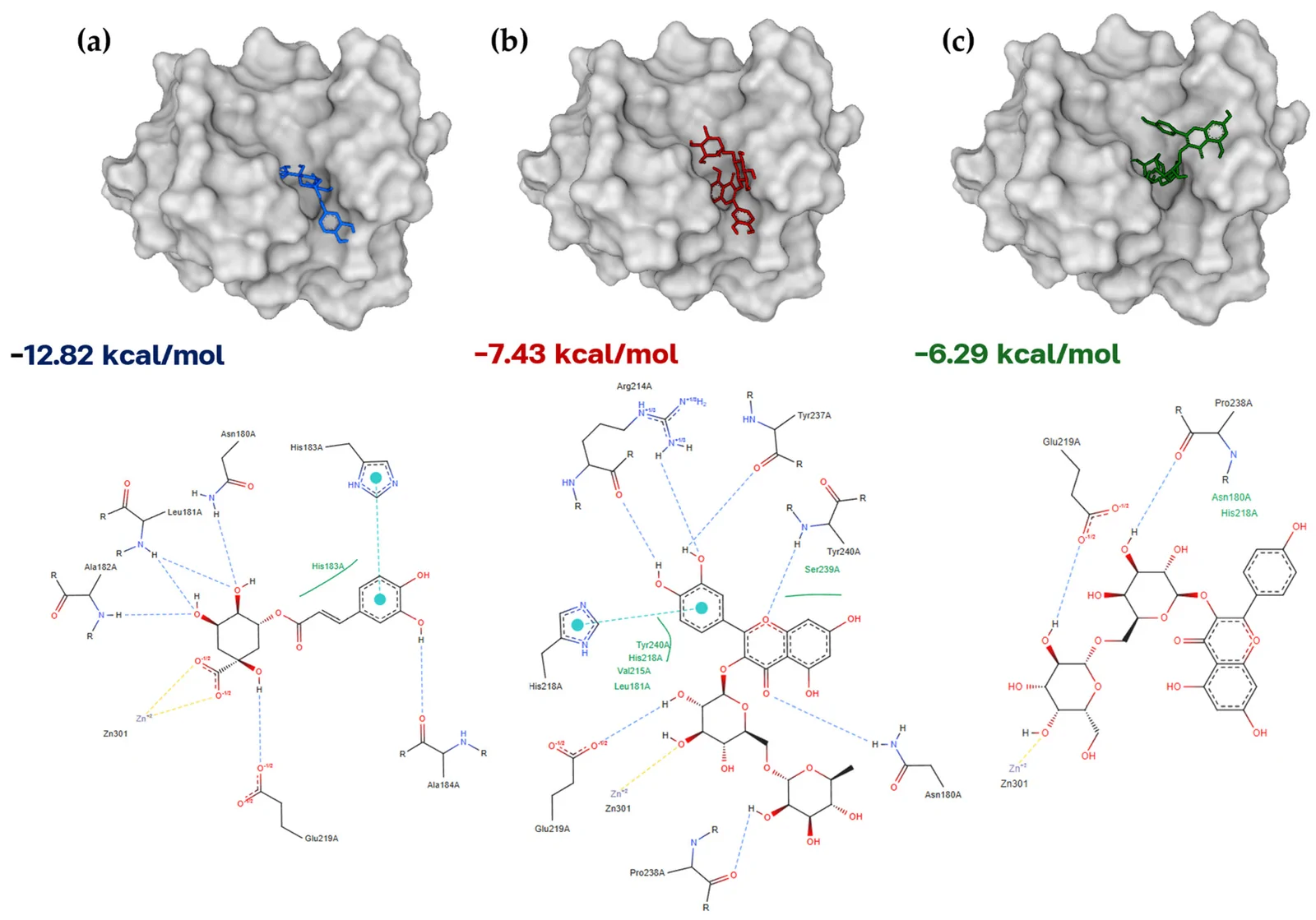
Binding conformations of (**a**) chlorogenic acid, (**b**) rutin, and (**c**) K3G within the collagenase active site. Ligands and key interacting residues are presented in stick format; 2D interaction diagrams were generated using PoseView, where hydrogen bonds are indicated by blue dashed lines, metal interactions by yellow dashed lines, π–π interactions by cyan dashed lines, and hydrophobic contacts by green solid lines.

The anti-collagenase potential of MLE was determined in terms of potential binding conformation through a molecular docking approach. The results demonstrated that the identified compounds in the MLE, including chlorogenic acid, rutin, and K3G, exhibited binding energies against collagenase of −12.82, −7.43, and −6.29 kcal/mol, respectively. Notably, all three compounds showed binding energies lower than −6.0 kcal/mol, indicating strong binding affinity toward the target protein [18]. All three compounds interacted within the catalytic pocket, showing metal-interacting contacts with the catalytic Zn^2+^ ion (Zn301), along with hydrogen bonding and hydrophobic interactions. Chlorogenic acid exhibited the most extensive interaction network, forming hydrogen bonds with Asn180A, Leu181A, Ala182A, Glu219A, and Ala184A, a π–π interaction between its catechol ring and His183A, a hydrophobic contact with Ala184A, and metal coordination with Zn301. Rutin also coordinated with Zn301 and formed hydrogen bonds with Arg214A, Tyr237A, Tyr240A, Asn180A, Pro238A, and Glu219A, together with a π–π interaction between its catechol (B-ring) moiety and His218A, and hydrophobic contacts with His218A, Tyr240A, Ser239A, Leu181A, and Val215A. Notably, both chlorogenic acid and rutin engaged the active site through π–π stacking of their respective catechol rings, whereas K3G, which lacks a catechol group, displayed hydrogen bonding with Glu219A and Pro238A and metal interaction with Zn301, but no comparable aromatic-stacking interaction within the catalytic core. Overall, chlorogenic acid exhibited the lowest binding energy and the most extensive interaction profile among the tested compounds, consistent with its markedly stronger predicted binding affinity (Figure 5).

## 3. Discussion

From the optimization results of UAE, the significant final models were successfully established, and the effects of the independent variables on the studied dependent variables could be interpreted as follows.

### 3.1. Effects of Variables on Extraction Yield

The significant positive effect of the liquid-to-solid ratio (A) on extraction yield is consistent with the principle that increasing solvent volume enhances mass transfer by maintaining a greater concentration gradient between the plant matrix and the surrounding solvent [19]. Similarly, the ultrasound amplitude (C) positively influenced extraction yield through enhanced cavitation intensity and mechanical cell disruption, which facilitated the release of intracellular compounds into the solvent [20]. However, the negative quadratic term of the amplitude (C^2^) indicates diminishing returns at higher power levels, beyond which further increases in amplitude did not translate into additional improvements in extraction yield. This behavior can be attributed to a cavitation saturation effect, in which excessive bubble formation at high ultrasonic intensity leads to inter-bubble collisions and the formation of a bubble layer, resulting in less effective bubble collapse and hindered energy transmission [21]. In addition, the negative effects of the AC and BC interaction terms suggested that operating at high amplitude simultaneously with high solvent volume or prolonged extraction time did not provide synergistic benefits; instead, the system appeared to reach a steady state under these combined conditions. From a practical standpoint, optimization should therefore balance extraction efficiency against solvent consumption, energy input, and processing time, rather than maximizing any single variable in isolation.

### 3.2. Effects of Variables on Phytochemical Recovery

Although higher solvent volume initially promotes mass transfer, the negative linear and quadratic effects of A on TPC and TFC suggested that excessive solvent volume beyond the optimum level led to dilution of phenolic compounds in the crude extract, likely due to the co-extraction of non-target ballast substances [22]. A similar phenomenon was reported by Nguyen et al. [23], who demonstrated that increasing solvent volume beyond a threshold enhanced the co-extraction of non-target components and consequently reduced both TPC and TFC concentrations in peanut skin extracts. Additionally, the negative effect of the AC interaction supported this interpretation, indicating that simultaneously increasing solvent volume and ultrasound intensity compounded the dilution effect rather than improving phenolic recovery. Notably, the significant positive quadratic effect of amplitude (C^2^) on TPC and TFC suggests that ultrasound power played a key role in enhancing phenolic and flavonoid extraction through cavitation-induced cell wall disruption, which facilitated phenolic diffusion into the solvent, consistent with the findings of Chotphruethipong et al. [24] in cashew leaf extraction.

In contrast to TPC, TFC exhibited a significant positive BC interaction, indicating that prolonged extraction time combined with appropriate ultrasound amplitude is essential for effective flavonoid recovery. This behavior is likely attributable to the predominance of glycosylated flavonoid derivatives in *M. speciosa* leaves [25], which, as previously reported, tend to be sequestered within plant vacuoles via membrane transport mechanisms [26]. Such intracellular compartmentalization restricts mass transfer between the target compounds and the extraction solvent, rendering passive diffusion alone insufficient for complete flavonoid liberation. Acoustic cavitation generated during UAE provides the mechanical force necessary to disrupt cell wall integrity and enhance solvent penetration into subcellular compartments, thereby facilitating the release of vacuole-stored flavonoid glycosides, a mechanism directly supported by Lai et al. [27] who demonstrated a 32% reduction in residual cellulose content and an 8.5% increase in flavonol glycoside yield from *Ginkgo biloba* following UAE-induced cell wall disruption relative to passive solvent extraction. The significance of the BC interaction, therefore, reflects the complementary roles of extended contact time and acoustic disruption in accessing compartmentalized flavonoids.

### 3.3. Effects of Variables on Antioxidant Activity

In both IC_50_ and EC_1_ models, where lower values reflect greater antioxidant capacity, the positive quadratic term A^2^ indicated that excessive solvent volume diminished antioxidant capacity, mirroring the dilution-driven reduction in phenolic concentration described above, whereas the negative quadratic term B^2^ confirmed that prolonged extraction time progressively improved antioxidant performance in both assays, consistent with more complete recovery of bioactive compounds over time. The ultrasound amplitude (C) contributed to IC_50_ reduction exclusively through the negative AC and BC interaction terms, with no significant effect observed for EC_1_, suggesting that cavitation modulates radical scavenging capacity in a condition-dependent manner rather than acting as an independent driver. These trends closely corresponded with the variations in TPC and TFC observed under the same extraction conditions, consistent with the findings of Anantaworasakul et al. [28], who reported that the radical scavenging capacity of *M. speciosa* leaf extracts is primarily attributed to their phenolic and flavonoid constituents.

Although acoustic cavitation has been reported to generate hydroxyl radicals capable of inducing oxidative degradation of phenolic compounds, such degradation follows a dose-response relationship and occurs only when hydroxyl radical concentration exceeds the compound-specific response threshold [29]. The absence of a significant negative main effect of the amplitude on either IC_50_ or EC_1_ in the present study suggests that cavitation within the amplitude range investigated did not compromise the antioxidant capacity of the extract, in contrast to the pronounced diminishing effect of excessive solvent volume reflected through the A^2^ term, which represents a purely physical dilution phenomenon. This interpretation is supported by Peng et al. [30], who demonstrated in a propolis extraction system that mild ultrasound conditions not only preserved polyphenol types and functional group integrity but also enhanced DPPH and ABTS radical scavenging activity, indicating that moderate cavitation contributes positively to antioxidant performance rather than undermining it. Collectively, these findings suggest that the amplitude conditions applied in the present study favored phytochemical release while remaining below the threshold at which sonochemical degradation becomes detrimental to antioxidant capacity.

### 3.4. Phytochemical Composition and Antioxidant Activity of MLE

Antioxidant activity has been recognized as an important protective mechanism in anti-aging strategies. In addition to scavenging ROS and alleviating oxidative stress–induced cellular damage, antioxidant activity contributes to collagen preservation by modulating enzymatic processes involved in collagen degradation [31].

Several compounds identified in the present study, including mitragynine, chlorogenic acid, rutin, and kaempferol derivatives, are consistent with previous reports on *M. speciosa* leaves [6,25,32,33,34], supporting the reproducibility of the phytochemical profile. Variations in detected constituents compared with earlier reports may be attributed to differences in geographical origin, environmental conditions, and extraction parameters [35]. Although alkaloids were the most abundant class, antioxidant activity is more commonly attributed to phenolic acids and flavonoids due to their hydroxyl-rich structures [5,36]. Our findings through Pearson’s correlation analysis confirmed that TFC, rather than TPC, was the significant contributor to the antioxidant capacity of MLE. However, the TFC assay is not entirely flavonoid-specific, as the AlCl_3_-only step is selective for flavonols, whereas the preceding NaNO_2_ step also reacts with catechol-bearing non-flavonoid phenolics such as chlorogenic acid [37]. The TFC signal in MLE therefore likely reflects a combined contribution from flavonols (e.g., rutin and kaempferol derivatives) and catechol-bearing phenolics like chlorogenic acid, explaining why TFC exceeded TPC in this study. Flavonoid content, together with chlorogenic acid, may therefore be a more reliable marker of MLE’s antioxidant potential than total phenolic content alone.

### 3.5. Human Fibroblast Safety and Anti-Collagenase Enzyme Property

The consistently high cell viability of MLE-treated NHDF indicates that the extract did not reduce cell viability within the tested concentration range, supporting an acceptable safety profile for potential topical application. Beyond this safety consideration, collagen accounts for approximately three-quarters of the dry weight of human skin and represents the most prevalent component of the dermal extracellular matrix [38]. Elevated MMP-1 (collagenase-1) activity degrades this collagen network and directly contributes to the deterioration of skin integrity [39], and collagenase has therefore been identified as a predominant strategy in functional anti-aging research targeting the delay of skin aging.

Although chlorogenic acid exhibited the strongest predicted binding energy against collagenase in the in silico study, its inhibitory potency in vitro did not differ significantly from that of rutin. This finding suggests that the two catechol-bearing markers contribute comparably to the anti-collagenase activity of the extract, rather than the activity being driven predominantly by a single compound. Discordance between predicted binding affinity and measured potency is not without precedent, even under matched experimental and computational conditions. Employing the same docking protocol and in vitro assay system, Wang et al. [40] reported that bavachalcone displayed a stronger predicted binding score against elastase than isobavachalcone (−2.421 vs. −1.559 kcal/mol), yet isobavachalcone proved markedly more potent in the corresponding in vitro assay (IC_50_ = 0.79 vs. 10.56 µM). Such findings support interpreting the present docking results as supportive, exploratory evidence rather than confirmation of a specific mechanism.

Furthermore, when the quantified content of chlorogenic acid and rutin in MLE is considered alongside their individual IC_50_ values, the two markers appear insufficient to fully account for the extract’s overall anti-collagenase activity. Additional constituents may therefore contribute, including kaempferol 3-glucosyl-(1→6)-galactoside (K3G), which coordinated the catalytic Zn^2+^ ion in the in silico study, as well as alkaloids such as mitragynine, recently reported to exhibit moderate collagenase inhibition activity (69.9 ± 3.7%) at 1 mg/mL [28]. As K3G was neither isolated nor quantified, and its tentative (Level 3) assignment and Zn^2+^ coordination remain unconfirmed, its contribution remains hypothetical. Given that mitragynine was quantified at an appreciable level in the MLE (approximately 5.9% *w*/*w*), it may plausibly contribute to the overall anti-collagenase activity of the extract. Nevertheless, direct evaluation of the respective contributions of these compounds was beyond the scope of the present study, which was designed to investigate phenolic and flavonoid constituents as the primary indicators of anti-collagenase potential. Accordingly, further studies are required to clarify the specific roles of mitragynine and K3G in collagenase inhibition.

However, the collagenase inhibitory activity of MLE remained lower than that of the pure reference compounds, including L-ascorbic acid, chlorogenic acid, and rutin. This is expected because MLE is a crude extract, in which the bioactive constituents are present at lower concentrations than in their purified forms. Therefore, the present findings should be interpreted as preliminary evidence supporting the anti-collagenase potential of MLE rather than demonstrating potent anti-aging efficacy. Nevertheless, these results suggest that MLE possesses promising anti-collagenase activity, highlighting its potential as a source of natural anti-aging constituents. Although mitragynine itself may contribute to the observed activity, its associated toxicity and controlled status necessitate removal of the alkaloid fraction before application, as discussed in the following section. Future development of MLE for anti-aging applications should therefore focus on its phenolic and flavonoid constituents, which can be retained while the alkaloid fraction is removed. Since skin aging is a multifactorial process, further studies evaluating additional targets, including hyaluronidase and elastase inhibition as well as intracellular reactive oxygen species (ROS) levels, are warranted to provide a more comprehensive assessment of the anti-aging potential of MLE.

### 3.6. Cosmeceutical and Nutraceutical Applicability of MLE

Beyond its established role as an anti-collagenase marker discussed above, chlorogenic acid has independently been documented for a broad range of nutraceutical and cosmeceutical bioactivities. As a nutraceutical, it exhibits antioxidant, anti-inflammatory, antilipidemic, antidiabetic, and antihypertensive properties, with in vivo and clinical evidence supporting its efficacy in ameliorating components of metabolic syndrome, including obesity, dyslipidemia, insulin resistance, and hepatic steatosis [41]. In cosmeceutical applications, chlorogenic acid displays strong free-radical-scavenging and UV-filtering activity, along with anti-tyrosinase and anti-inflammatory effects that support its use as a photoprotective and hyperpigmentation-correcting ingredient [42]. The remaining principal flavonoid constituents identified in MLE collectively suggest broader nutraceutical applicability. Rutin has been documented for its antioxidant and anti-inflammatory properties, with evidence demonstrating radical scavenging activity and modulation of pro-inflammatory mediators including TNF-α, IL-6, NO, and PGE2 [43]. Similarly, kaempferol 3-glucosyl-(1→6)-galactoside, a glycosylated derivative of kaempferol, may potentially exhibit bioactivities consistent with those reported for kaempferol and related glycosides, including antioxidant, anti-inflammatory, anticancer, and cardioprotective effects, based on class-level analogy rather than direct evidence for K3G in this extract [44]. Collectively, the antioxidant and anti-inflammatory activities shared among chlorogenic acid, rutin, and kaempferol glycosides support the potential of MLE as a topical cosmeceutical targeting oxidative stress and photoaging, while the additional cardioprotective and metabolic health benefits reported for kaempferol glycosides and chlorogenic acid further support the nutraceutical potential of these constituents for future incorporation into oral products.

Nevertheless, as *M. speciosa* leaves inherently contain mitragynine, a psychoactive indole alkaloid that acts as a partial opioid receptor agonist, its consumption has been associated with dose-dependent hepatotoxicity, neurotoxicity, and addiction potential, with human case reports documenting cholestatic liver injury, seizures, and even fatal outcomes [4]. Consequently, any nutraceutical formulation derived from this plant would require strict control of alkaloid content in accordance with the varied regulatory frameworks governing mitragynine across countries and product categories [45]. In the present study, mitragynine was quantified at approximately 5.9% *w*/*w*, confirming that it was co-extracted at an appreciable level rather than excluded. Because mitragynine itself is scheduled as a controlled substance in several jurisdictions (e.g., Australia, Sweden, and Malaysia) irrespective of concentration, the presence of mitragynine at this level would limit the direct application of the current MLE in regulated nutraceutical and cosmeceutical markets. A dedicated purification step, such as acid–base partitioning or solid-phase extraction to remove or substantially reduce the alkaloid fraction, would therefore be a necessary prerequisite before the phenolic and flavonoid constituents of MLE could be developed into consumer products. Nevertheless, while the current extract would require isolation or removal of mitragynine before application, this study opens a new perspective on *M. speciosa* by highlighting its non-mitragynine constituents, demonstrating that the phenolic and flavonoid fraction represents an underexplored reservoir of cosmeceutical and nutraceutical bioactivity for future valorization.

## 4. Materials and Methods

### 4.1. Materials

All standard compounds and reagents were purchased from Sigma-Aldrich (Burlington, MA, USA) unless otherwise stated, including L-ascorbic acid, quercetin, gallic acid, chlorogenic acid, rutin, mitragynine, Folin–Ciocalteu reagent, 2,2-diphenyl-1-picrylhydrazyl (DPPH), 2,4,6-tris(2-pyridyl)-s-triazine (TPTZ), 3,4-dihydroxyphenylacetic acid (DHPAA), bovine collagen, and collagenase. Ferrous sulfate (FeSO_4_) was obtained from LOBA Chemie (Mumbai, India). Cell culture reagents, including Dulbecco’s Modified Eagle Medium (DMEM), fetal bovine serum (FBS), penicillin (100 U/mL), streptomycin (100 mg/mL), and sulforhodamine B (SRB), were procured from Thermo Fisher Scientific (Waltham, MA, USA). Ethanol (95% *v*/*v*) was purchased from L Pure Natural (Chiang Mai, Thailand). All other chemicals were of analytical grade. The NHDF cells were provided by Manose Health and Beauty Research Center Co., Ltd. (Chiang Mai, Thailand).

### 4.2. Plant Materials

*M*. *speciosa* leaves of the red-vein (Karn Daeng) variety were sourced from local farms in Phrae Province, Thailand during November 2021. All leaves were collected from the same cultivation site. The cultivation site in Phrae Province, northern Thailand, is located in a tropical savanna climate region (Köppen Aw), which is predominant in the area [46]. The plant material was botanically identified by comparison with the taxonomic descriptions reported by Puff et al. [47]. Botanical identification was performed by Wannaree Charoensup, a botanist at the Faculty of Pharmacy, Chiang Mai University, Chiang Mai, Thailand. A voucher specimen (No. 0023484) has been deposited at the Herbarium, Faculty of Pharmacy, Chiang Mai University, Chiang Mai, Thailand. The collected leaves were dried in a hot-air oven at 50 °C, ground into a fine powder, and stored at 4 °C until extraction.

### 4.3. M. speciosa Leaf Extraction

UAE was conducted using *M. speciosa* leaf powder mixed with 95% (*v*/*v*) ethanol as the solvent and performed with an ultrasonic processor (VibraCell VCX130, Sonics & Materials Inc., Newtown, CT, USA) equipped with a ½-inch diameter probe, under fixed operating conditions of 130 W power and 20 kHz frequency, with a pulse mode of 5 s on/off. After completion of the extraction, the obtained solution was filtered to remove residues, and the solvent was subsequently removed using a rotary evaporator (Rotavapor^®^ R-300, Büchi Labortechnik AG, Flawil, Switzerland) at 50 °C to obtain the crude extract. The weight of the crude extract was measured and calculated as the %Yield, as shown in the equation.

(6) $$\%\mathrm{yield}=\frac{\mathrm{Crude}\mathrm{extract}\mathrm{weight}\;(g)}{\mathrm{Dried}\mathrm{plant}\mathrm{weight}\;(g)}\times 100$$

### 4.4. Experimental Design of UAE Conditions for Extraction

The experimental design for optimizing UAE conditions was established based on a CCD. Three independent variables of the UAE, including liquid-to-solid ratio (mL/g; A), extraction time (min; B), and amplitude power (%; C), were systematically investigated to generate the optimal extraction conditions for dried *M. speciosa* leaf powder. The optimal condition should achieve the highest extraction yield (Y_1_), along with the highest phytochemical content, including total phenolic content (mg GAE/g; Y_2_) and total flavonoid content (mg QE/g; Y_3_), as well as the highest antioxidant potential evaluated by DPPH and FRAP assays, expressed as IC_50_ (Y_4_) and EC_1_ (Y_5_), respectively.

Each independent variable was assigned five levels according to the CCD, including low-axial, low, center, high, and high-axial levels, which were coded as −α, −1, 0, +1, and +α, respectively. The coded and actual levels of all variables are presented in Table 9. A total of 20 experimental runs were generated using Design-Expert^®^ software (version 10.0, Stat-Ease, Minneapolis, MN, USA). After completion of the experiments, the data were analyzed using analysis of variance (ANOVA), with the significance level at *p* < 0.05. Response surface plots and corresponding quadratic polynomial equations were then generated for each response (Equation (7)). For antioxidant activity responses (IC_50_ and EC_1_), the quadratic models were expressed in natural logarithmic form, as shown in Equation (8).

(7) $$y=\beta_{0}\;+\;\beta_{A}A\;+\;\beta_{B}B\;+\;\beta_{C}C\;+\;\beta_{\mathrm{AB}}\mathrm{AB}\;+\;\beta_{\mathrm{AC}}\mathrm{AC}\;+\;\beta_{\mathrm{BC}}\mathrm{BC}\;+\;\beta_{\mathrm{AA}}A^{2}\;+\;\beta_{\mathrm{BB}}B^{2}\;+\;\beta_{\mathrm{CC}}C^{2}$$

(8) $$\log_{10}\left(y\right)=\beta_{0}\;+\;\beta_{A}A\;+\;\beta_{B}B\;+\;\beta_{C}C\;+\;\beta_{\mathrm{AB}}\mathrm{AB}\;+\;\beta_{\mathrm{AC}}\mathrm{AC}\;+\;\beta_{\mathrm{BC}}\mathrm{BC}\;+\;\beta_{\mathrm{AA}}A^{2}\;+\;\beta_{\mathrm{BB}}B^{2}\;+\;\beta_{\mathrm{CC}}C^{2}$$

In addition, model verification was conducted using the optimized conditions under which the experimental values were compared with the predicted values derived from the model equations. Prediction accuracy was evaluated in terms of the percentage of relative standard error (%RSE) using Equation (9). The %RSE values less than 5% were acceptable [48].

(9) $$\%\mathrm{RSE}=\left|\frac{\mathrm{Predicted}\mathrm{value}-\mathrm{Actual}\mathrm{value}}{\mathrm{Predicted}\mathrm{value}}\right|\times 100$$

### 4.5. Determination of Phytochemical Content

#### 4.5.1. Total Phenolic Content (TPC)

The colorimetric reaction based on the Folin–Ciocalteu reagent method was used to determine the total phenolic content (TPC) of the extract. The absorbance of the reaction mixture was detected at 765 nm by a UV-vis spectrophotometer (UV2600i, Shimadzu, Kyoto, Japan). The absorbance was substituted into the linear equation of the gallic acid calibration curve. The result was expressed in units of milligrams of gallic acid equivalents per gram of extract (mgGAE/g).

#### 4.5.2. Total Flavonoid Content (TFC)

Briefly, the ethanolic sample solution was mixed with 10% *w*/*v* aluminium chloride solution, 5% *w*/*v* sodium nitrite, 1 M NaOH, and DI water. The mixture was then left at room temperature for 30 min, allowing a complete chemical reaction. Then, the absorbance of the solution was measured by a UV-vis spectrophotometer at 415 nm. TFC, in units of milligrams of quercetin equivalents per gram of extract (mgQE/g), was calculated by substituting the absorbance of the extract into the calibration curve equation of quercetin.

### 4.6. Phytochemical Profile Determined by UHPLC-ESI-QTOF-MS/MS

The phytochemical profile of the MLE was characterized using UHPLC-QTOF-MS. The analysis was performed on an Agilent 1290 Infinity II UHPLC system coupled to an Agilent 6545 LC-QTOF/MS, equipped with a reversed-phase C18 column. A binary gradient elution was employed, consisting of 0.1% (*v*/*v*) formic acid in water (solvent A) and acetonitrile (solvent B) at a flow rate of 0.2 mL/min. Mass spectrometric detection utilized a dual Agilent Jet Stream electrospray ionization (AJS-ESI) source in both positive and negative ion modes. Data were acquired in full-scan mode (*m*/*z* 100–1100) with Auto-MS/MS experiments conducted at collision energies of 10, 20, and 40 eV. Compounds were putatively identified by comparing accurate mass measurements, isotopic distributions, and MS/MS fragmentation patterns against established databases. This profiling guided the selection of key bioactive compounds for subsequent correlation analysis and molecular docking studies. Candidate compounds were matched against the MassHunter METLIN Metabolite PCD (Personal Compound Database) and PCDL (Personal Compound Database and Library), the latter incorporating experimental MS/MS reference spectra. Tentative identification was accepted only for candidates with a match score exceeding 80% and a mass error within ±5 ppm. The identification confidence level of each compound was subsequently classified according to the criteria of Schymanski et al. [17].

### 4.7. Quantification of Phytochemical Compounds by HPLC-UV

Quantification of chlorogenic acid and rutin in the MLE was performed using high-performance liquid chromatography with ultraviolet detection (HPLC-UV; Shimadzu LC-2030C, Shimadzu Corporation, Kyoto, Japan). All samples and standards were dissolved in methanol (MeOH); the MLE was analyzed at a concentration of 250 µg/mL and filtered through a 0.45 µm membrane filter prior to injection. Chromatographic separation was achieved on a Purospher^®^ STAR RP-18 column (250 × 4.6 mm, 5 µm) maintained at 40 °C, with an injection volume of 20 µL and a flow rate of 1.0 mL/min. The mobile phase consisted of 0.01% (*v*/*v*) phosphoric acid in water, adjusted to pH 2.7 (solvent A), and acetonitrile (solvent B), delivered as a gradient elution program: 10% B (0.01–12.00 min), a linear increase to 20% B (12.00–28.00 min), held at 20% B (28.00–30.00 min), a linear increase to 35% B (30.00–40.00 min), held at 35% B (40.00–50.00 min), a linear decrease to 10% B (50.00–60.00 min), and re-equilibration at 10% B (60.00–65.00 min). UV detection was performed at 350 nm. Chlorogenic acid and rutin were identified by comparison of retention times with those of authentic reference standards. Calibration curves were constructed using six concentration levels ranging from 1 to 100 µg/mL, with each concentration analyzed in triplicate (*n* = 3). The limit of detection (LOD) and limit of quantification (LOQ) were calculated as LOD = 3.3 × SD/S and LOQ = 10 × SD/S, respectively, where SD is the standard deviation of the y-intercept of the regression line and S is the slope of the calibration curve. Mitragynine was quantified separately using a different set of chromatographic conditions optimized for its analysis, with the corresponding method and chromatogram presented in the Supplementary Material (Supplementary Figure S2).

### 4.8. Antioxidant Evaluation

#### 4.8.1. DPPH Radical Scavenging Inhibition

To evaluate the antioxidant potential of the extract in terms of the hydrogen atom transfer (HAT) mechanism [49], the colorimetric reaction with DPPH at 520 nm was used and the absorbance of the reaction mixture was measured using a microplate reader (SPECTROstar, BMG Labtech, Ortenberg, Germany). The method was conducted following the study of Jaikampan et al. [50]. Serial concentrations of sample solution were diluted with absolute ethanol and mixed with 0.067 mg/mL of DPPH solution in a ratio of 1:10 to reach a final concentration of 3.125–100 µg/mL. Then, percentage of DPPH scavenging inhibition (%inhibition) was calculated using the following Equation (10). The graph of the relationship between %inhibition and concentration was plotted, then the concentration that expresses the 50% inhibition was reported as the 50% inhibitory concentration (IC_50_) value. In addition to MLE, chlorogenic acid and rutin, the quantified candidate marker compounds were evaluated using the same assay, with L-ascorbic acid serving as the positive control.

(10) $$\%\mathrm{inhibition}=\left(\frac{\left({\mathrm{Abs}}_{c}-{\mathrm{Abs}}_{\mathrm{bc}}\right)-\left({\mathrm{Abs}}_{s}-{\mathrm{Abs}}_{\mathrm{bs}}\right)}{\left({\mathrm{Abs}}_{c}-{\mathrm{Abs}}_{\mathrm{bc}}\right)}\right)\times 100$$

where Abs_s_ and Abs_c_ are the absorbances of the sample and control, respectively, while Abs_bs_ and Abs_bc_ are the absorbance of the sample blank and control blank.

#### 4.8.2. Ferric Reducing Antioxidant Power (FRAP)

The antioxidant potential in terms of single electron transfer (SET) was determined by using colorimetric reaction with ferric ion in FRAP solution [49], which contained 10 mM TPTZ in 40 mM HCl solution, 300 mM acetate buffer, and 20 mM FeCl_3_. Briefly, the FRAP solution was mixed with serial concentrations of sample extract ranging from 31.25–1000 µg/mL in ethanolic solution. Then the mixture was incubated in the dark for 30 min, and the absorbance of the mixture was measured at 593 nm. The graph of the relationship between absorbance and concentration was plotted, then the concentration which expresses the equivalent absorbance to 1 mM of FeSO_4_ was reported as the EC_1_ value. In addition to MLE, chlorogenic acid and rutin, the quantified candidate marker compounds were evaluated using the same assay, with L-ascorbic acid serving as the positive control.

### 4.9. Cytotoxicity on Human Dermal Fibroblasts

The NHDF cells were maintained in a culture medium supplemented with L-glutamine, 10% fetal bovine serum (FBS), and 1% penicillin–streptomycin and incubated at 37 °C in a humidified atmosphere containing 5% CO_2_.

The cytotoxicity of the extract toward NHDF cells was evaluated using the sulforhodamine B (SRB) assay according to the method of Vichai & Kirtikara [51] with slight modification. Briefly, the extract was dissolved in cell culture medium (DMEM supplemented with 10% FBS and 1% penicillin–streptomycin) containing 10% (*v*/*v*) DMSO, sterilized through a 0.2-µm membrane filter, and serially diluted to obtain the desired concentrations. NHDF cells were seeded in 96-well plates at a density of 1 × 10^5^ cells/well and allowed to attach for 24 h before treatment with the extract at final concentrations of 0.0001–1 mg/mL for 24 h at 37 °C under 5% CO_2_. The final DMSO concentration was maintained at 0.5% (*v*/*v*) in all treatments, and medium containing 0.5% (*v*/*v*) DMSO served as the vehicle control. After incubation, the adherent cells were fixed in situ, washed and dyed with SRB. The bound dye was solubilized, and the absorbance was measured at 540 nm. Cell viability was expressed as the percentage of absorbance relative to vehicle control according to Equation (11). L-Ascorbic acid and sodium lauryl sulfate (SLS) were included as negative and positive controls, respectively, while the culture medium served as the blank control.

(11) $$\%\mathrm{cell}\mathrm{viability}=\frac{{\mathrm{Abs}}_{s}}{{\mathrm{Abs}}_{c}}\times 100$$

where Abs_s_ is the absorbance of the tested sample and Abs_c_ is the absorbance of the control.

### 4.10. Collagenase Enzyme Inhibition Evaluation

The anti-collagenase activity of the MLE was assessed according to the method reported by Sirirungsee et al. [52] with minor modifications. In brief, the sample was serially diluted using 20% (*w*/*v*) Polysorbate 20 in water and combined with a reaction mixture consisting of 10 mM calcium chloride (CaCl_2_), 125 mM borate buffer (pH 7.5), and collagenase enzyme. The mixture was incubated at 37 °C in the dark for 60 min, followed by the addition of the collagen substrate and a fluorescence reagent containing 0.75 mM DHPAA and 1.25 mM sodium periodate (NaIO_4_). The reaction was then incubated for an additional 10 min. The final sample concentrations ranged from 7.81 to 125 µg/mL, with 20% (*w*/*v*) Polysorbate 20 serving as the control. Fluorescence intensity was recorded using a microplate reader (SpectraMax M3, Molecular Devices, San Jose, CA, USA) at excitation and emission wavelengths of 375 nm and 465 nm, respectively. Collagenase inhibition was expressed as a percentage, and the concentration–inhibition relationship was used to calculate the IC_50_ value. In addition to MLE, chlorogenic acid and rutin, the quantified candidate marker compounds were evaluated using the same assay, with L-ascorbic acid serving as the positive control.

### 4.11. Molecular Docking Analysis

The selected polyphenol compounds identified in the MLE, including chlorogenic acid, kaempferol 3-glucosyl-(1→6)-galactoside, and rutin, were subjected to molecular docking analysis. 3D-structures of the selected compounds were retrieved from the PubChem database (accessed on 15 December 2025), pre-optimized using Avogadro [53], and subsequently optimized at the quantum mechanical level using the ORCA program version 6.1 [54,55] with the B97-3c composite method [56]. The collagenase enzyme structure (PDB ID: 1CGL) was obtained from the RCSB Protein Data Bank [57]. Prior to docking, co-crystallized ligands and water molecules were removed, and the protein structure was prepared using AutoDockTools version 1.5.7 by adding polar hydrogens. Partial charges were assigned using the Gasteiger method for the ligand and the Kollman method for the protein, and non-polar hydrogens were merged prior to docking. Molecular docking simulations were then performed using AutoDock 4 [58] with the Lamarckian Genetic Algorithm (LGA), with the grid box defined to cover the active site of the enzyme. Ligand–protein interactions were subsequently analyzed and visualized using Discovery Studio Visualizer 2021 [59] and PoseView accessed via the ProteinsPlus web server (University of Hamburg, Hamburg, Germany; accessed on 20 December 2025) [60].

### 4.12. Statistical Analysis

All experiments were conducted in triplicate, and the results were expressed as the mean ± standard deviation (SD). Statistical analyses were performed using SPSS software (version 17.0 for Windows; IBM Corp., Armonk, NY, USA). Pearson’s correlation analysis was applied to evaluate the relationships between TPC and TFC with antioxidant activities expressed as IC_50_ and EC_1_, respectively, with the correlation coefficients interpreted according to the criteria reported by Dancey & Reidy [61]. For comparison of biological activities (antioxidant and anti-collagenase activities) among MLE and the reference/marker compounds, one-way analysis of variance (ANOVA) followed by Tukey’s post hoc test was performed. Statistical significance was defined at *p* < 0.05.

## 5. Conclusions

This study provides preliminary evidence for the cosmeceutical potential of *M. speciosa* leaf extract, highlighting the value of its phenolic and flavonoid constituents as an underexplored alternative to the alkaloid profile that has traditionally defined this plant. Systematic UAE optimization yielded an extract with high polyphenol content and strong antioxidant performance, with rutin and chlorogenic acid identified as the principal contributors. The optimized extract showed measurable collagenase inhibitory activity while maintaining high fibroblast viability, indicating sufficient preliminary potential to warrant further development as an anti-aging ingredient. Molecular docking identified chlorogenic acid as the strongest predicted collagenase binder, though this remains supportive, exploratory evidence, as chlorogenic acid and rutin together could not fully account for the extract’s activity, pointing to yet-unidentified contributing constituents. As skin aging is multifactorial, complementary assessment of other anti-aging aspects such as hyaluronidase, elastase, and intracellular ROS is needed to more comprehensively substantiate this potential. Importantly, mitragynine was found to be co-extracted at an appreciable level (approximately 5.9% *w*/*w*), and its associated toxicity and controlled status mean that strict control or removal of the alkaloid fraction is a prerequisite before the extract can be considered suitable for cosmeceutical or nutraceutical application. Collectively, this study offers the first integrated investigation of the phenolic and flavonoid constituents of *M. speciosa*, establishing new groundwork for its cosmeceutical and nutraceutical potential, while its practical applicability remains contingent on further studies addressing alkaloid content control and safety.

## Figures and Tables

**Table 1:** Central Composite experimental design of UAE parameters for optimization of extracting *Mitragyna speciosa* leaves.

Run	Type	Independent Variables			Dependent Variable				
		LS Ratio (mL/g, A)	Time (min, B)	Amplitude (%, C)	%Yield (%, Y_1_)	TPC (mgGAE/g, Y_2_)	TFC (mgQE/g, Y_3_)	IC_50_ DPPH (µg/mL, Y_4_)	EC_1_ FRAP (µg/mL, Y_5_)
1	Axial	6 (0)	23.4 (+α)	40 (0)	9.92	172.57	355.79	23.92	495.47
2	Center	6 (0)	15 (0)	40 (0)	8.6	176.20	366.51	37.47	730.92
3	Center	6 (0)	15 (0)	40 (0)	9.12	179.83	355.79	50.96 *	871.82
4	Axial	6 (0)	15 (0)	57 (+α)	7.74	238.53	546.65	38.75	723.23
5	Factorial	8 (+1)	10 (−1)	30 (−1)	7.63	191.33	415.83	38.83	782.67
6	Factorial	8 (+1)	20 (+1)	50 (+1)	9.49	167.72	396.53	32.34 *	668.79
7	Factorial	4 (−1)	10 (−1)	50 (+1)	8.51	194.35	338.63	28.11 *	892.64
8	Factorial	8 (+1)	10 (−1)	50 (+1)	9.92	174.99	334.34	37.08	788.83
9	Center	6 (0)	15 (0)	40 (0)	8.72	162.28	355.79	41.18	819.09
10	Axial	9.37 (+α)	15 (0)	40 (0)	10.63	112.65	205.67	47.83	951.50 *
11	Axial	6 (0)	6.6 (−α)	40 (0)	8.56	182.85	334.34	35.10	867.18 *
12	Axial	6 (0)	15 (0)	23 (−α)	7.24 *	148.96 *	409.40 *	33.74	724.15
13	Factorial	4 (−1)	10 (−1)	30 (−1)	6.75 *	175.59	432.99	36.15	873.55
14	Axial	2.63 (−α)	15 (0)	40 (0)	5.11	147.75	291.45 *	77.41	1588.17
15	Center	6 (0)	15 (0)	40 (0)	9.08	179.22	366.51	45.25	955.00
16	Factorial	4 (−1)	20 (+1)	50 (+1)	8.72	191.93	353.64	38.74	793.83
17	Center	6 (0)	15 (0)	40 (0)	8.12	183.74	385.81	34.78	954.80
18	Center	6 (0)	15 (0)	40 (0)	8.02	185.88	349.35	40.17	798.00
19	Factorial	4 (−1)	20 (+1)	30 (−1)	4.29	166.51	317.19	43.75	953.20
20	Factorial	8 (+1)	20 (+1)	30 (−1)	8.48	170.75	362.22	50.63	798.00

* Responses marked with (*) indicate missing independent variable (outliers).

**Table 2:** ANOVA results and statistical evaluation of quadratic response surface models for *M. speciosa* leaf extraction optimization.

Terms	Factor Codes	F-Value				
		%Yield (Y_1_)	TPC (Y_2_)	TFC (Y_3_)	IC_50_ (Y_4_)	EC_1_ (Y_5_)
Linear terms	A	162.22 ^a^	6.02 ^a^	1.16	20.18 ^a^	5.54 ^a^
	B	9.52 ^a^	2.81	0.44	11.43 ^a^	0.68
	C	104.13 ^a^	1.18	2.86	0.01	1.03
Quadratic terms	$A^{2}$	5.02	50.61 ^a^	43.87 ^a^	40.85 ^a^	31.69 ^a^
	$B^{2}$	5.27	0.011	0.05	16.84 ^a^	28.74 ^a^
	$C^{2}$	68.31 ^a^	33.36 ^a^	85.08 ^a^	-	5.17
Interaction terms	AB	2.15	0.39	2.85	-	0.215
	AC	30.18 ^a^	6.03 ^a^	0.03	17.53 ^a^	0.01
	BC	5.72 ^a^	0.59	14.5 ^a^	20.95 ^a^	2.44
R^2^		0.9696	0.9279	0.9448	0.9149	0.9283
Adjusted R^2^		0.9354	0.8551	0.8826	0.8487	0.8476
Predicted R^2^		0.8864	0.7236	0.7441	0.7770	0.8054
Adequate precision		21.84	17.79	19.98	18.55	17.73
Regression (*p*-value)		<0.0001 ^a^	0.0004 ^a^	0.0004 ^a^	0.0004 ^a^	0.0011 ^a^
Lack of fit (*p*-value)		0.8292	0.7627	0.6503	0.6246	0.9002

^a^ Indicates significant terms at *p* < 0.05; TPC: total phenolic content. TFC: total flavonoid content.

**Table 3:** The predicted value and actual value of responses under optimized conditions (*n* = 3).

Responses	Predicted Value	Actual Value	% RSE
%Yield (%, Y_1_)	9.34	9.60 ± 0.39	2.78
TPC (mg GAE/g, Y_2_)	195.07	188.53 ± 2.21	3.35
TFC (mg QE/g, Y_3_)	439.63	426.67 ± 18.98	2.95
IC_50_ (µg/mL, Y_4_)	23.63	24.77 ± 1.17	4.82
EC_1_ (µg/mL, Y_5_)	612.01	633.51 ± 50.67	3.51

**Table 4:** Pearson’s correlation of phytochemical compounds and antioxidant potential of the MLE (*n* = 23).

**TPC**	**IC_50_**	**EC_1_**
Pearson’s Correlation	−0.249	−0.331
Sig (2-tailed)	0.253	0.123
Correlated Definition	Not Correlated	Not Correlated
**TFC**	**IC_50_**	**EC_1_**
Pearson’s Correlation	−0.491 *	−0.502 *
Sig (2-tailed)	0.017	0.015
Correlated Definition	Moderate correlation	Moderate correlation

* *p* < 0.05; correlations with *p* > 0.05 are considered non-significant.

**Table 5:** Phytochemical profile identified in optimized MLE extract by UHPLC-ESI-QTOF-MS/MS.

Compound	RT (min)	Molecular Formula	*m*/*z* (ion)	Mass (Experimental)	Error (ppm)	Match Score	Confidence Level
**Alkaloids**							
Cinegalline	17.636	C_23_H_30_N_2_O_6_	431.2176/(M+H)+	430.2104	0.02	96.62	Level 3
Desacetylvindoline	18.159	C_23_H_30_N_2_O_5_	415.2229/(M+H)+	414.2155	−0.08	97.79	Level 3
Rhynchophylline	18.165	C_22_H_28_N_2_O_4_	367.2021/(M+H)+[-H_2_O]	384.2054	−0.52	97.77	Level 3
Mitragynine	18.172	C_23_H_30_N_2_O_4_	399.2285/(M+H)+	398.2212	−0.64	94.47 *	Level 2a
(+)-Prosopinine	25.035	C_18_H_35_NO_3_	318.2404/(M+Na)+[-H_2_O]	313.2616	0.06	85.45	Level 3
**Flavonoids**							
Rutin	16.219	C_27_H_30_O_16_	609.1455/(M-H)-	610.1527	1.2	96.85 *	Level 2a
Kaempferol 3-glucosyl-(1→6)-galactoside	16.276	C_27_H_30_O_16_	633.1434/(M+Na)+	610.1535	−0.11	89.18	Level 3
**Phenolic acids**							
Chlorogenic acid	9.845	C_16_H_18_O_9_	353.0873/(M-H)-	354.0946	1.35	89.54 *	Level 2a
**Triterpenoids**							
Ceanothic acid	17.196	C_30_H_46_O_5_	469.3308/(M+H)+[-H_2_O]	486.3343	0.26	99.01	Level 3
**Glycosides**							
Isopropyl apiosylglucoside	10.916	C_14_H_26_O_10_	377.142/(M+Na)+	354.1529	−0.26	98.81	Level 3
**Sphingosides**							
6-hydroxysphingosine	25.794	C_18_H_37_NO_3_	320.2558/(M+Na)+[-H_2_O]	315.2772	0.15	99.22	Level 3
**Organic acids**							
Phosphoglycolic acid	0.481	C_2_H_5_O_6_P	154.9739/(M-H)-	155.9811	1.26	91.59	Level 3
Quinic acid	1.84	C_7_H_12_O_6_	191.056/(M-H)-	192.0632	0.83	82.18 *	Level 2a

Match scores were obtained from the accurate-mass PCD, except for asterisked (*) values, which were obtained from the PCDL incorporating MS/MS spectral matching.

**Table 6:** HPLC linear regression characteristics of chlorogenic acid, rutin, and mitragynine.

Compound	RT (min)	Linear Equation	R^2^	LOD (µg/mL)	LOQ (µg/mL)
Chlorogenic acid	9.32	y = 26,302x − 2078.8	0.9997	0.505	1.530
Rutin	28.33	y = 36,199x + 7034.1	0.9995	0.149	0.451
Mitragynine	3.60	y = 22,128x − 98,003	0.9991	0.071	0.214

**Table 7:** Quantified contents of chlorogenic acid, rutin, and mitragynine in the MLE (*n* = 3).

Compound	Retention Time (min)	Content (µg/mg Extract)
Chlorogenic acid	9.30 ± 0.05	31.37 ± 2.41
Rutin	28.32 ± 0.01	18.76 ± 1.66
Mitragynine	3.60 ± 0.06	59.38 ± 0.21

**Table 8:** Biological activity of MLE, Chlorogenic acid, Rutin, and L-ascorbic acid (*n* = 3).

Compound	IC_50_ DPPH (µg/mL)	EC_1_ FRAP (µg/mL)	IC_50_ Collagenase Inhibition (µg/mL)
MLE	24.77 ± 1.17 ^a^	633.51 ± 50.67 ^a^	92.63 ± 7.81 ^a^
Chlorogenic acid	5.30 ± 0.06 ^b^	148.49 ± 1.05 ^b^	32.23 ± 3.13 ^b^
Rutin	4.66 ± 0.06 ^b^	238.27 ± 6.69 ^c^	21.54 ± 1.08 ^b,c^
L-ascorbic acid	6.05 ± 0.35 ^b^	117.38 ± 2.56 ^b^	12.88 ± 0.26 ^c^

Different superscript letters indicate a statistically significant difference (*p* < 0.05).

**Table 9:** Optimization of UAE conditions of *M. speciosa* leaves by CCD.

Factors	Independent Variable Code				
	−α	−1	0	+1	+α
Liquid-Solid ratio (mL/g, A)	2.6	4	6	8	9.4
Extraction Time (min, B)	6.6	10	15	20	23.4
Amplitude (%, C)	23	30	40	50	57

## Data Availability

The raw data supporting the conclusions of this article will be made available by the authors upon request.

## Supplementary Materials

The following supporting information can be downloaded at: https://www.mdpi.com/article/10.3390/plants15162416/s1, Table S1. ANOVA parameter of model statistics comparison between original and adjust model (outlier exclusion). Figure S1. Predicted value and actual value plot from (a) original model without outlier elimination and (b) adjust model with outlier elimination. Table S2. Phytochemical profile identified in optimized MLE extract by UHPLC-ESI-QTOF-MS/MS. Figure S2. HPLC chromatograms of mitragynine standards at concentrations of (a) 10 µg/mL, (b) 30 µg/mL, and (c) 50 µg/mL, together with (d) MLE at a concentration of 500 µg/mL. Supplementary Methods. Quantification of mitragynine by HPLC-UV.

## Abbreviations

The following abbreviations are used in this manuscript:

AJS-ESI	Agilent Jet Stream electrospray ionization
CCD	Central composite design
DPPH	2,2-diphenyl-1-picrylhydrazyl
EC_1_	Equivalence concentration of 1mM FeSO_4_
FRAP	Ferric reducing antioxidant power
HAT	Hydrogen atom transfer
HPLC	High-Performance Liquid Chromatography
IC_50_	Inhibit concentration at 50%
K3G	Kaempferol 3-glucosyl-(1→6)-galactoside
LC-QTOF/MS	Liquid Chromatography–Quadrupole Time-of-Flight Mass Spectrometry
LOD	Limit of detection
LOQ	Limit of quantification
mgGAE/g	Milligrams of gallic acid equivalents per gram of extract
mgQE/g	Milligrams of quercetin equivalents per gram of extract
MLE	Optimized *M. speciosa* leaf extract
MMPs	Matrix metalloproteinases
MS/MS	Tandem mass spectrometry
NHDF	Normal human dermal fibroblast
PCD	Personal Compound Database
PCDL	Personal Compound Database and Library
ROS	Reactive oxygen species
SET	Single electron transfer
TFC	Total flavonoid content
TPC	Total phenolic content
UAE	Ultrasound-assisted extraction
UHPLC-QTOF	Ultra-high-performance liquid chromatography coupled with quadrupole time-of-flight
%RSE	Percentage of relative standard error
