# Supplementary material for: Optimized Ultrasound-Assisted Extraction Enhances the Recovery of Anti-Collagenase Phytochemicals from Mitragyna speciosa Korth. Leaves
Source: Plants (Basel). 2026 Aug 7;15(16):2416. doi: 10.3390/plants15162416 (PMC13516103; doi:10.3390/plants15162416)
Supplement: Supplementary file 1 [file plants-15-02416-s001.zip › plants-4409519-supplementary.pdf]

**Table S1.** ANOVA parameter of model statistics comparison between original and adjust model (outlier exclusion)

| ANOVA parameter | %Yield ($Y_1$) | | TPC ($Y_2$) | | TFC ($Y_3$) | | $IC_{50}$ DPPH ($Y_4$) | | $EC_1$ FRAP ($Y_5$) | |
|---|---|---|---|---|---|---|---|---|---|---|
| | **Original** | **Adjust** | **Original** | **Adjust** | **Original** | **Adjust** | **Original** | **Adjust** | **Original** | **Adjust** |
| Model (*p*-value) | 0.0219 | <0.0001 | 0.0545 | 0.0004 | 0.0295 | 0.0004 | 0.2536 | 0.0004 | 0.0075 | 0.0011 |
| Model $R^2$ | 0.7798 | 0.9696 | 0.7293 | 0.9279 | 0.7833 | 0.9448 | 0.4680 | 0.9149 | 0.8281 | 0.9283 |
| Adjusted $R^2$ | 0.5815 | 0.9354 | 0.4780 | 0.8551 | 0.5883 | 0.8826 | 0.1577 | 0.8487 | 0.6734 | 0.8476 |
| Predicted $R^2$ | -0.5951 | 0.8864 | -0.7953 | 0.7236 | -0.4259 | 0.7441 | -0.7974 | 0.7770 | 0.0869 | 0.8054 |
| Adeq Precision | 7.0517 | 21.84 | 7.7313 | 17.79 | 8.5024 | 19.98 | 5.1707 | 18.55 | 10.8261 | 17.73 |
| Lack of fit (*p*-value) | 0.0185 | 0.8292 | 0.0604 | 0.7627 | 0.0552 | 0.6503 | 0.3175 | 0.6246 | 0.2804 | 0.9002 |

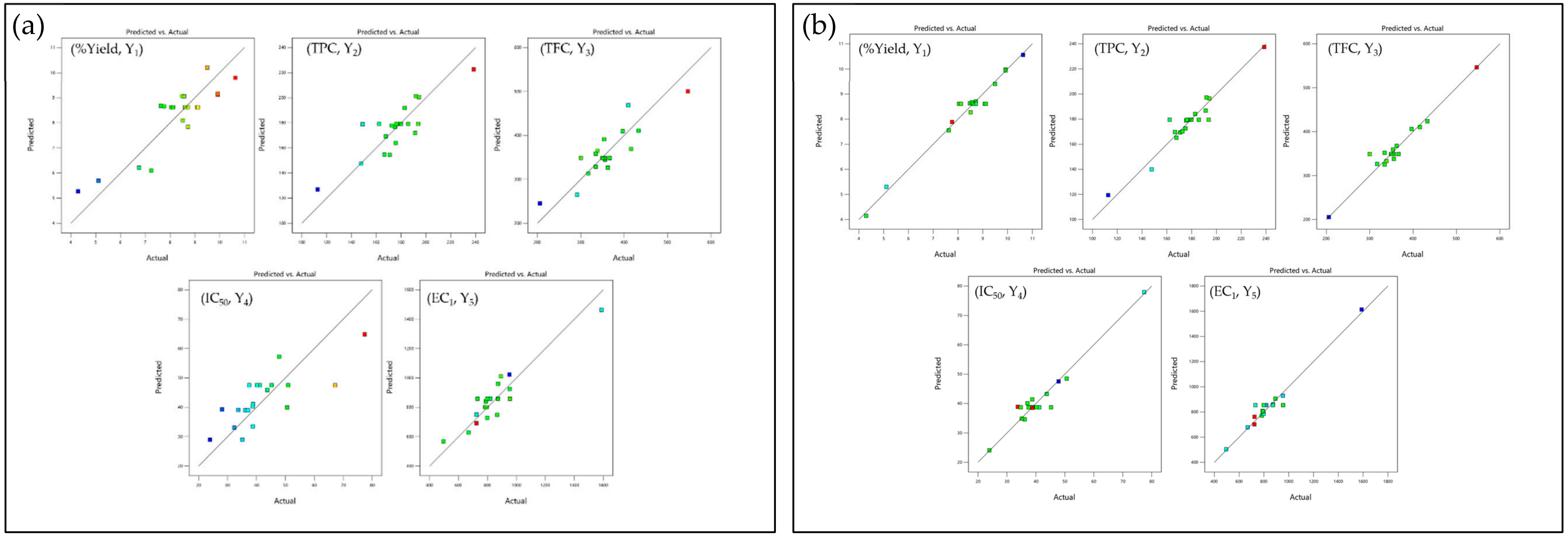


**Figure S1**. Predicted value and actual value plot from (a) original model without outlier elimination and (b) adjust model with outlier elimination

**Table S2.** Phytochemical profile identified in optimized MLE extract by UHPLC-ESI-QTOF-MS/MS

| Compound | RT (min) | Molecular Formula | m/z, ions | Characteristic MS/MS fragments | Mass (Experimental) | Error (ppm) | Match Score (PCD) | Match Score (PCDL) | Confidence Level |
|---|---|---|---|---|---|---|---|---|---|
| Mitragynine | 18.172 | C23 H30 N2 O4 | 399.2285 (M+H)+ | 174.0913, 175.0965, 226.1438, 227.143, 238.1438, 367.2016, 382.2013 | 398.2212 | -0.64 | 97.96 | 94.47 | Level 2a |
| Rhynchophylline | 18.165 | C22 H28 N2 O4 | 367.2021 (M+H)+[-H2O] | 55.0542, 153.0546, 197.1073, 367.2016, 368.2048 | 384.2054 | -0.52 | 97.77 | - | Level 3 |
| Desacetylvindoline | 18.159 | C23 H30 N2 O5 | 415.2229 (M+H)+ | 99.0804, 190.0893, 217.0972, 240.1594, 271.1414, 383.1965 | 414.2155 | -0.08 | 97.79 | - | Level 3 |
| Kaempferol 3-glucosyl-(1->6)-galactoside | 16.276 | C27 H30 O16 | 633.1434 (M+Na)+ | 85.0284, 407.082 | 610.1535 | -0.11 | 89.18 | - | Level 3 |
| Isopropyl apiosylglucoside | 10.916 | C14 H26 O10 | 377.142 (M+Na)+ | 174.0311, 319.1387 | 354.1529 | -0.26 | 98.81 | - | Level 3 |
| Ceanothic acid | 17.196 | C30 H46 O5 | 469.3308 (M+H)+[-H2O] | 263.1642, 287.2006, 335.2733, 451.3207 | 486.3343 | 0.26 | 99.01 | - | Level 3 |
| Cinegalline | 17.636 | C23 H30 N2 O6 | 431.2176 (M+H)+ | 82.0651, 158.9951, 238.1465, 239.1496 | 430.2104 | 0.02 | 96.63 | - | Level 3 |
| (+)-Prosopinine | 25.035 | C18 H35 N O3 | 318.2404 (M+Na)+[-H2O] | 55.0178, 95.0855, 188.0832, 216.1019, 238.0413 | 313.2616 | 0.06 | 85.45 | - | Level 3 |
| 6-hydroxysphingosine | 25.794 | C18 H37 N O3 | 320.2558 (M+Na)+[-H2O] | 53.0542, 102.1277, 194.109, 205.0886, 218.0937 | 315.2772 | 0.15 | 99.22 | - | Level 3 |
| Chlorogenic acid | 9.845 | C16 H18 O9 | 353.0873 (M-H)- | 191.0561, 192.0595, 353.0878 | 354.0946 | 1.35 | 99.17 | 89.54 | Level 2a |
| Rutin | 16.219 | C27 H30 O16 | 609.1455 (M-H)- | 129.0546, 303.0499, 304.0533, 420.9826, 465.1028, 466.1062 | 610.1527 | 1.2 | 98.51 | 96.85 | Level 2a |
| Quinic acid | 1.84 | C7 H12 O6 | 191.056 (M-H)- | 55.0189, 57.0346, 59.0139, 69.0346, 71.0139, 85.0295, 93.0346 | 192.0632 | 0.83 | 99.76 | 82.18 | Level 2a |
| Phosphoglycolic acid | 0.481 | C2 H5 O6 P | 154.9739 (M-H)- | 66.9954, 110.9853, 111.988, 154.9751 | 155.9811 | 1.26 | 91.59 | - | Level 3 |

**Supplementary Methods. Quantification of mitragynine by HPLC-UV**

Because of its pharmacological activity and safety relevance, mitragynine was quantified in the MLE by HPLC-UV (Shimadzu LC-2030C) under conditions optimized separately from those used for the phenolic and flavonoid constituents. Standards and MLE at a concentration of 500 µg/mL (in methanol, filtered 0.45 µm) were separated on a ZORBAX Eclipse Plus C18 column (150 × 4.6 mm, 3.5 µm) at 35 °C, 10 µL injection, 1.2 mL/min, using acetonitrile (A) and 0.1% formic acid in water (B): 30% A (0 min) to 70% A (1 min), held to 4 min, returned to 30% A (6 min), and re-equilibrated to 10 min. Detection was at 254 nm, with identification against an authentic standard. Calibration used six levels (1–50 µg/mL, triplicate).

Under these conditions, two main peaks were observed in the mitragynine standard chromatograms (Supplementary Figure S2). The peak at 3.60 min was assigned to mitragynine, as its area increased proportionally with concentration across the calibration range and its retention time matched that of the authentic standard. In contrast, the peak at approximately 7.4 min remained relatively constant in intensity regardless of mitragynine concentration, indicating that it was concentration-independent and therefore unrelated to the analyte. This peak is likely attributable to a system or gradient-related signal arising from the mobile-phase composition change during the elution program, rather than to a co-eluting compound.

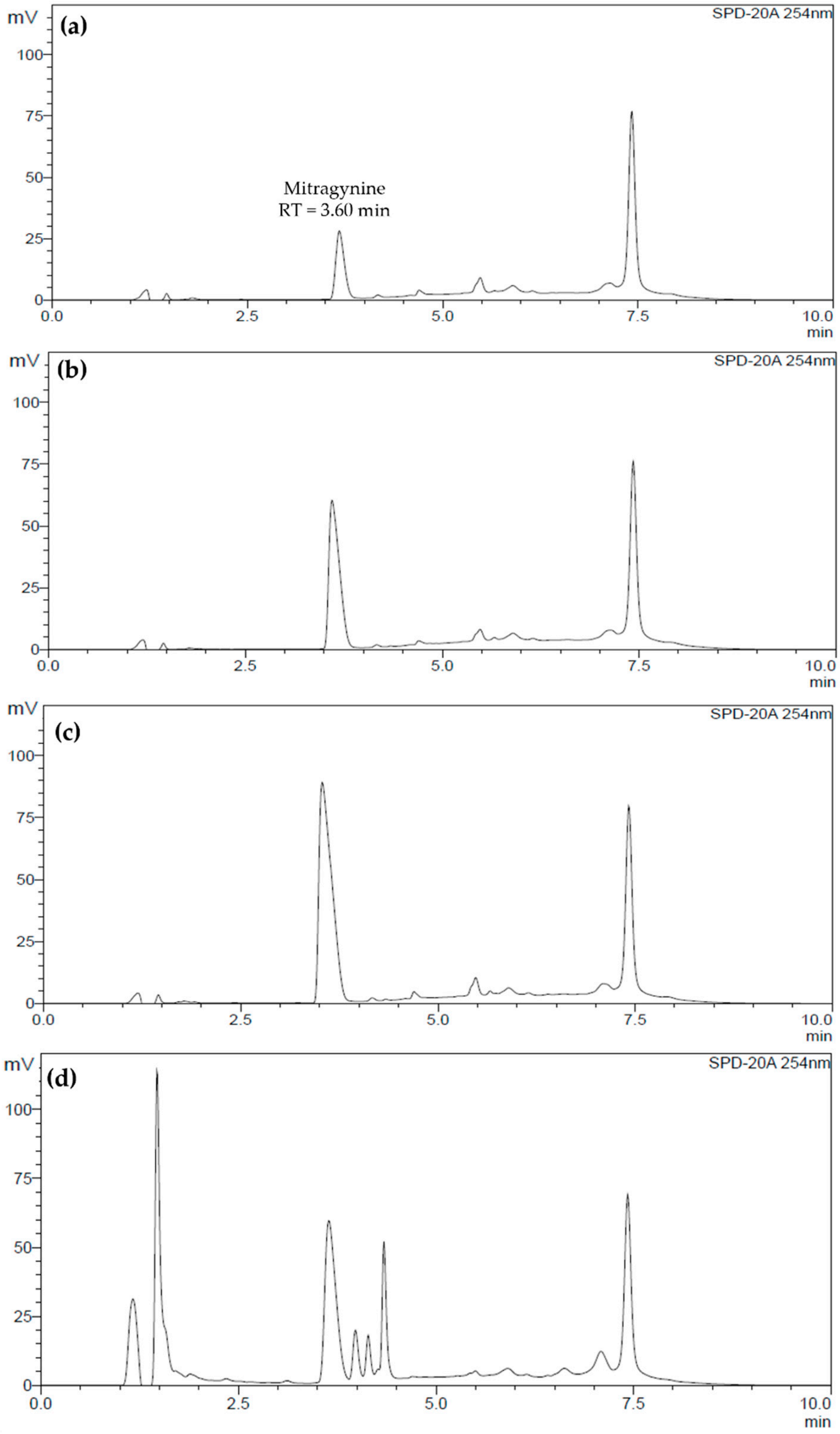

**Figure S2**. HPLC chromatograms of mitragynine standards at concentrations of (a) 10 μg/mL, (b) 30 μg/mL, and (c) 50 μg/mL, together with (d) MLE at a concentration of 500 μg/mL.
